# A comprehensive map of the influenza A virus replication cycle

**DOI:** 10.1186/1752-0509-7-97

**Published:** 2013-10-02

**Authors:** Yukiko Matsuoka, Hiromi Matsumae, Manami Katoh, Amie J Eisfeld, Gabriele Neumann, Takeshi Hase, Samik Ghosh, Jason E Shoemaker, Tiago JS Lopes, Tokiko Watanabe, Shinji Watanabe, Satoshi Fukuyama, Hiroaki Kitano, Yoshihiro Kawaoka

**Affiliations:** 1 JST ERATO Kawaoka infection-induced host responses project, Minato-ku, Tokyo 108-8639, Japan; 2 The Systems Biology Institute, Minato-ku, Tokyo 108-0071, Japan; 3 Department of Bioinformatics, Medical Research Institute, Tokyo Medical and Dental University, Bunkyo-ku, Tokyo 113-8540, Japan; 4 Department of Pathological Science, School of Veterinary Medicine, University of Wisconsin-Madison, Madison, WI 53711, USA; 5 Laboratory of Veterinary Microbiology, Department of Veterinary Sciences, University of Miyazaki, Miyazaki 889-2192, Japan; 6 Sony Computer Science Laboratories, Inc., Shinagawa, Tokyo 141-0022, Japan; 7 Okinawa Institute of Science and Technology Graduate University, Onna-son, Okinawa 904-0495, Japan; 8 Division of Virology, Department of Microbiology and Immunology, The Institute of Medical Science, The University of Tokyo, Minato-ku, Tokyo 108-8639, Japan; 9 Department of Special Pathogens, International Research Center for Infectious Diseases, Institute of Medical Science, University of Tokyo, Minato-ku, Tokyo 108-8639, Japan; 10 Laboratory of Bioresponses Regulation, Department of Biological Responses, Institute for Virus Research, Kyoto University, Kyoto 606-8507, Japan

**Keywords:** Drug targets, FluMap, Host factors, Influenza virus, Pathways

## Abstract

**Background:**

Influenza is a common infectious disease caused by influenza viruses. Annual epidemics cause severe illnesses, deaths, and economic loss around the world. To better defend against influenza viral infection, it is essential to understand its mechanisms and associated host responses. Many studies have been conducted to elucidate these mechanisms, however, the overall picture remains incompletely understood. A systematic understanding of influenza viral infection in host cells is needed to facilitate the identification of influential host response mechanisms and potential drug targets.

**Description:**

We constructed a comprehensive map of the influenza A virus (‘IAV’) life cycle (‘FluMap’) by undertaking a literature-based, manual curation approach. Based on information obtained from publicly available pathway databases, updated with literature-based information and input from expert virologists and immunologists, FluMap is currently composed of 960 factors (i.e., proteins, mRNAs etc.) and 456 reactions, and is annotated with ~500 papers and curation comments. In addition to detailing the type of molecular interactions, isolate/strain specific data are also available. The FluMap was built with the pathway editor CellDesigner in standard SBML (Systems Biology Markup Language) format and visualized as an SBGN (Systems Biology Graphical Notation) diagram. It is also available as a web service (online map) based on the iPathways+ system to enable community discussion by influenza researchers. We also demonstrate computational network analyses to identify targets using the FluMap.

**Conclusion:**

The FluMap is a comprehensive pathway map that can serve as a graphically presented knowledge-base and as a platform to analyze functional interactions between IAV and host factors. Publicly available webtools will allow continuous updating to ensure the most reliable representation of the host-virus interaction network. The FluMap is available at http://www.influenza-x.org/flumap/.

## Background

Rapid adaption to new hosts and frequent antigenic alterations make the prevention and treatment of influenza A virus (IAV) infections challenging. To develop better intervention methods, a deeper understanding of the viral infection process and the host response to infection are critical. IAV possesses an RNA genome of ~12 kilobases (kb) that encodes 10–12 proteins. As a consequence of this small coding capacity, IAVs usurp and modify the host cell machinery to replicate. Several studies have now provided extensive datasets on cellular factors that may directly or indirectly affect the viral life cycle [1-6] (works are reviewed in [7,8]). However, it has been challenging to integrate and compare this information with other published data, and to develop a complete picture of the viral life cycle. To this end, a comprehensive illustration and annotation of the current knowledge of the IAV infection process with underlying textual descriptions would greatly assist in elucidating the mechanisms by which influenza viruses utilize host cell machinery and evade host defence mechanisms.

Interaction networks, such as protein-protein interaction (PPI) networks, are often used to visualize interactions among entities (for example, proteins), but such networks do not capture the directionality of interactions (for example, “who stimulates whom”). In addition, interaction networks typically do not capture interactions between different types of molecules (for example, protein–RNA interactions). For these purposes, pathway visualization approaches, that is, ‘pathway maps’ – such as those described for Epidermal Growth Factor Receptor (EGFR) [9], Toll-like receptor (TLR) [10,11], retinoblastoma protein/E2F (Rb/E2F) [12], yeast [13], or mammalian target of rapamycin (mTOR) [14] – are better suited. Furthermore, while a graphical representation provides the best overview of biological phenomena, it is also important to represent the model in a machine-readable format that can be rigorously analysed using *in silico* methods.

Several projects have generated open-source, open-access databases of viral genome sequences, structural and interaction data for viral proteins, and host response data (e.g., the Influenza Research Database [15], the Influenza Virus Resource [16], and VirusMINT [17]); or pathway maps of IAV infections (e.g., Reactome [18,19] and KEGG [20]). Among the available pathway maps, the ‘Influenza A’ KEGG map contains only a limited number of entities and reactions. A greater amount of detail is available in the Reactome ‘Influenza Life Cycle’ and ‘Host Interactions with Influenza Virus Factors’ maps; however, these maps have not been updated since their creation in 2006, and the lack of integration between them makes it difficult to obtain insights into how they are interrelated. Both the KEGG and Reactome maps also lack significant additional information about pathway entities (e.g., PubMed IDs, supportive references) and neither is readily amenable to computational analysis approaches unless their pathways are converted to standard file formats that can be imported to analytic tools such as Cytoscape. Therefore, the usefulness of both the KEGG and the Reactome pathways as information- and hypothesis-generating platforms is limited.

**Figure 1:**
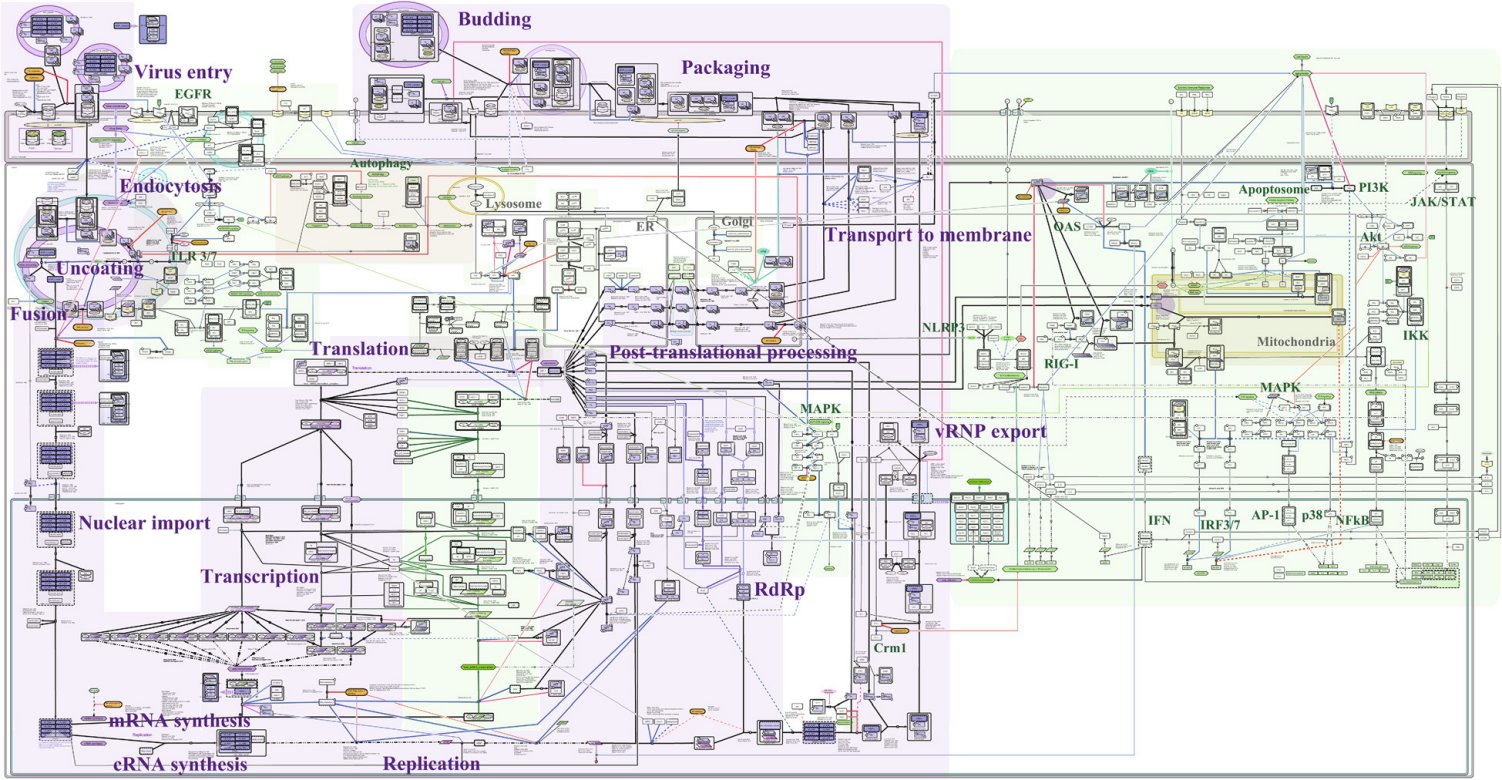
**FluMap, a comprehensive IAV pathway map.** FluMap was created with CellDesigner version 4.3. A total of 960 factors and 456 reactions were included. The SBML and high-resolution image PDF files are available as Additional Data. When FluMap is opened in CellDesigner, all factors, reactions, and cellular compartments included in the map are listed in the SBML file, and symbols used to build the map are illustrated in the legend of Additional file 3. (See also Additional file 2).

To address these shortcomings and improve our understanding of influenza virus infections, we created an integrated, comprehensive and interactive map that includes both viral life cycle and host response processes (i.e., the “FluMap”) (Figure 1). Here, we describe the FluMap construction strategy, highlight some of the map’s major characteristics, and demonstrate how it can be used as a bioinformatics tool. FluMap will be made available at a website (http://www.influenza-x.org/flumap) and can be used in conjunction with the online curation platform Payao [21] and a pathway browsing platform iPathways+ [22]. Together, these tools enable the scientific community to freely and simultaneously browse, add, and update FluMap information, thus providing the foundation for a powerful, community-curated knowledge base to further influenza virus research.

## Construction and contents

### Construction of a comprehensive, knowledge-based pathway map of influenza virus infection (FluMap)

The information used to build the FluMap (Figure 1; Additional file 1, Additional file 2, Additional file 3, and Additional file 4) was derived from several different sources. First, we manually reconstructed the Reactome ‘Influenza Life Cycle’ and ‘Host Interactions with Influenza Virus Factors’ maps [18,19] into a single map file (the FluMap pathway ‘skeleton’). Next, we manually incorporated information about host pathways that are activated in response to influenza virus infection, and - for all validated interaction partners of IAV factors - we included information about downstream signalling and processing events (e.g., phosphorylation cascades). Host factor and pathway data were obtained by using published pathway maps, KEGG [20], PANTHER [23] and/or Reactome [18,19] pathway map databases. Finally, we manually integrated literature-based information regarding the influenza virus replication cycle and virus-host interactions that was absent from the Reactome pathway ‘skeleton’ (Approximately 13% of the interactions in the map were derived from the “skeleton”, and another 10% were collected from the public pathway databases). This information was identified from review articles, extensive searches on PubMed, and text-mining platforms such as iHOP [24].

Although recent siRNA screens [2-4,6], protein-protein interaction studies [5,25-28] and global proteome analyses [29,30] have identified a substantial number of cellular factors with potential roles in the IAV infection process, FluMap includes only those with roles that have been experimentally confirmed. In addition, FluMap focuses on *intracellular* events, and does not include intercellular events (e.g., immune cell interactions). All curated reactions and interactions in the FluMap were categorized into specific parts of the influenza infection process (e.g., ‘vRNP export), and for reactions imported from Reactome, we kept the reaction name from this database (e.g., ‘Entry of Influenza Virion into Host Cell via Endocytosis’). A similar naming strategy was used for other reactions manually added to the map (Additional file 2 and Additional file 5).

To build the graphical representation of the FluMap (Figure 1; Additional file 2, Additional file 3, and Additional file 4), we used CellDesigner ver.4.3 [31], a modeling software that can be used to depict cellular processes step-by-step, edit annotations, and provide links to reference databases [32]; we also used Payao, a community-based, collaborative web service platform for gene-regulatory and biochemical pathway model curation [21]. The map is stored in the standard Systems Biology Markup Language (SBML) (Additional file 4), a data exchange format based on XML [33]; and it is represented in the CellDesigner’s graphical notation [34], which adheres to the Systems Biology Graphical Notation (SBGN) standards [35]. Map graphics were produced using SBGN ‘process description’ language (Additional file 2), which allows for visualization of state transitions (e.g., stimulation or inhibition events). By using standard formats, we have enabled FluMap to be adaptable to multiple network analysis tools such as Cytoscape or to simulation by employing user-supplied kinetic laws and SBML-compliant simulators.

In addition to a detailed visual representation, we generated comprehensive, text-based annotations, which are stored in the same map file. CellDesigner enables annotation of information in three different ways: (1) in the Notes section; (2) in the MIRIAM (*M*inimum *I*nformation *R*equired *I*n the *A*nnotation of *M*odels) [36] format section; and (3) in an additional layer overlaying the base model. For FluMap, we used all three annotation options to maximise data accessibility (see Additional file 2 for details). Gene IDs, UniProt accession numbers, PubMed (reference) IDs, and Reactome IDs are stored in the Notes and MIRIAM sections. The Notes section also includes information about the intracellular location of specific interactions or reactions (e.g., ‘Nucleus’ or ‘Mitochondria’), the stage of the infection process at which it occurs (e.g., ‘Virus Entry’ or ‘vRNP Export’), the participation of specific viral proteins, and association with multi-protein complexes that regulate host processes (e.g., ‘Apoptosome’) or signalling pathways (e.g., ‘MAPK’). Additional reference information (e.g., ‘HA1: Yoshida R *et al.* 2009’) is captured in the layer that overlays the base model. CellDesigner provides direct access to the relevant databases mentioned in the Notes section through the CellDesigner database menu, and the weblinks in the MIRIAM section by pressing the access button.

**Figure 2:**
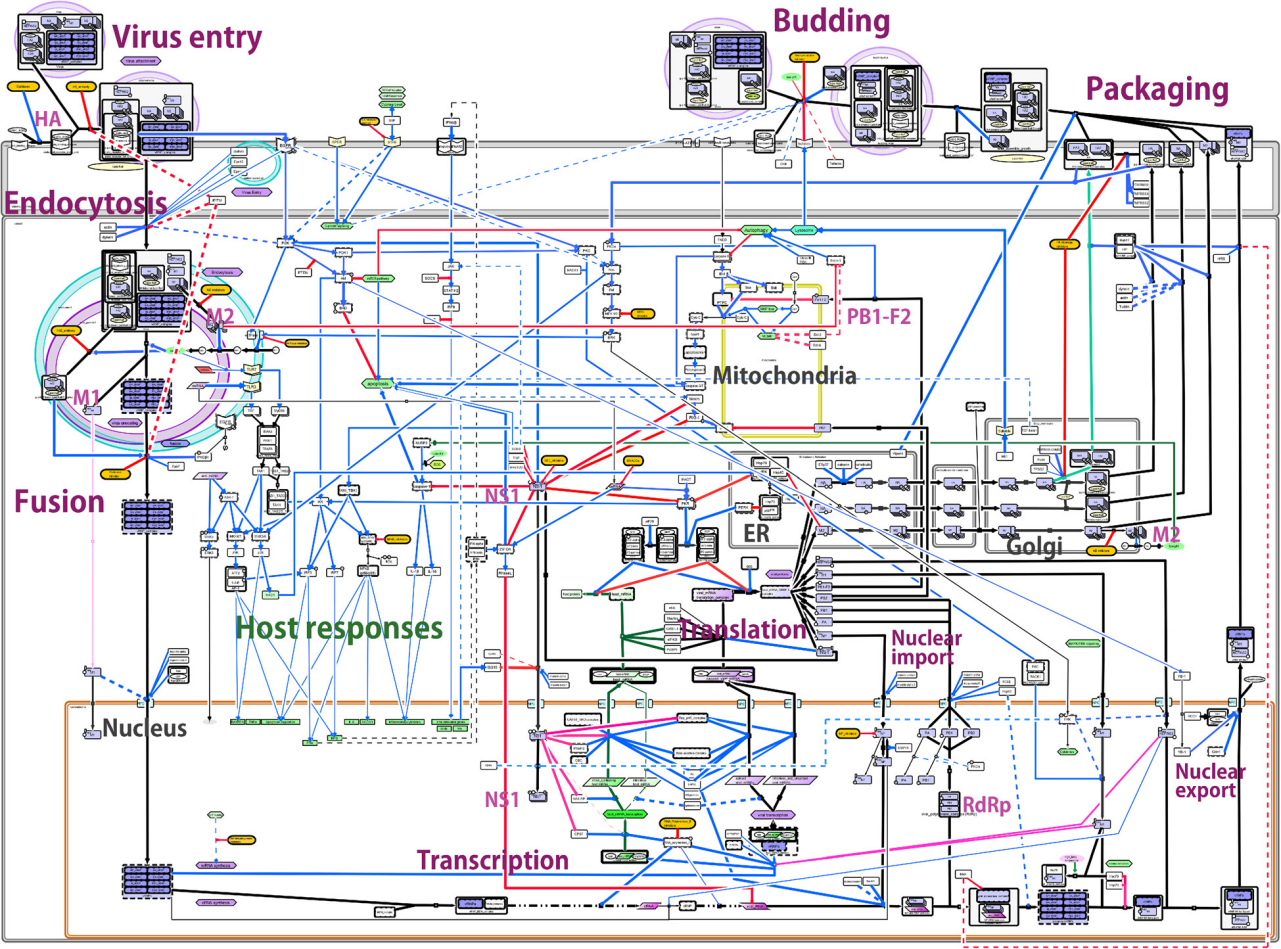
**Simplified version of FluMap.** To generate a simplified version of FluMap for a high-level overview, we extracted central components and reactions from the FluMap (virus factors (purple), host factors (green), antiviral factors (orange)), focusing on the inhibition (red) or activation (blue) of IAV replication by host factors.

While process description diagrams capture all details of biological processes, it is also useful to have a simplified overview of the system. We, therefore, used the ‘reduced notations’ option in CellDesigner to illustrate the relationships between entities (positive/negative inferences, modulation, trigger, etc.). This notation depicts positive/negative influence interactions, rather than detailed events, such as phosphorylation or catalysis in the process description notation (see Additional file 2 sections B and C). Finally, we used this notation to manually construct a simplified map (Figure 2; compare to the fully detailed FluMap in Figure 1) that provides a high-level overview of the IAV replication cycle.

The FluMap is posted under http://www.influenza-x.org/flumap, where users can browse its contents using a pathway-browsing platform (iPathways+) and provide updates and improvements using a manual curation platform (Payao).

### General characteristics of FluMap

The comprehensive FluMap (Figure 1; see Additional file 4 for the original SBML file) contains 960 factors (696 species + 264 factors hidden in complexes) and 456 reactions. Among these, there are 558 viral and cellular proteins, 212 molecular complexes (composed of more than one component), 12 ions, 55 ‘phenotypes’ (representing biological events such as apoptosis or autophagy), and 18 antiviral compounds. As described, all reactions are annotated with PubMed IDs in the Notes section; the entire map is annotated with 476 papers (Additional file 5 and Additional file 6). FluMap thus provides a significant improvement over the Reactome influenza infection pathway, which included 156 species and 58 reactions as of April 2012.

**Figure 3:**
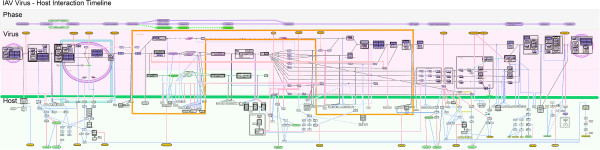
**Flowchart of the IAV life cycle.** The simplified FluMap (Figure 2) was converted into a linear process flow diagram and separated into the different phases of the viral life cycle (top portion), viral processes (middle portion), and host interaction factors (lower portion). Interactions are classified as inhibitory (red), stimulating (blue), and transition (black). Enclosure in the middle (orange line) indicates nucleoplasm.

While the FluMap adopts the SBGN’s process description graphical notation, the simplified map (Figure 2; Additional file 7) adopts the ‘reduced notation’ similar to SBGN’s activity flow, which better facilitates visualization of the virus-host interplay at different stages of the virus life cycle. To better highlight the virus-host interplay, we manually restructured the simplified FluMap into a linear flowchart that is divided into viral and host response events (Figure 3; Additional file 8). In this representation, it is easier to track the different phases of the viral life cycle (entry, endocytosis, transcription/translation, assembly, and budding).

### Description of the IAV replication cycle

In the following sections, we summarize our current knowledge of the IAV replication process as outlined in the FluMap (Figure 1), focusing on virus-host interactions.

#### Virus entry

The first step in the IAV life cycle is virus binding to host cells (‘Virus Entry’ , Figure 1). The viral hemagglutinin (HA) protein is critical for this step since it binds to sialic acids on host cell glycoproteins or glycolipids. The HA proteins of human IAVs preferentially recognize sialic acid linked to galactose by an α2,6-linkage (Siaα2,6Gal) [37-42] that is predominant on epithelial cells in the human upper respiratory tract [43-49]. In contrast, avian virus HA proteins preferentially bind to Siaα2,3Gal [37-42], which is predominantly found on epithelial cells of the duck intestine (where avian influenza viruses replicate) [39,50-52]. These differences in HA receptor specificity are a critical determinant of IAV host range (reviewed in [53-55]).

#### Endocytosis

Following receptor binding, IAVs enter cells through receptor-mediated endocytosis (‘Endocytosis’ in Figure 1). Clathrin-mediated endocytosis appears to be the primary internalization pathway of IAVs [56]; however, clathrin-independent endocytosis [57,58] and macropinocytosis [59,60] have also been described for IAV internalization. Several host factors including the small GTPases Rab5 and Rab7 [61], and interferon-inducible transmembrane IFITM protein family members (i.e., IFITM1, IFITM2, IFITM3) interfere with IAV internalization [1,62].

#### Fusion

At the low pH of the late endosome, HA undergoes an irreversible conformational shift which expels the N-terminus of the HA2 subunit (the so-called ‘fusion peptide’) so that it can insert into the endosomal membrane, resulting in the fusion of the viral and endosomal membranes (reviewed in [63]) (‘Fusion’ in Figure 1). Through an ion channel formed by the viral M2 protein, proton influx also acidifies the interior of the virus particles, leading to the dissociation of the viral matrix protein (M1) from viral ribonucleoprotein (vRNP) complexes [64]. vRNPs are composed of one of the eight viral RNAs (vRNAs), which are wrapped around the nucleoprotein (NP) and are also associated with the viral polymerase complex (see below). Dissociation from M1 allows vRNP release into the cytoplasm and subsequent nuclear import, which is mediated by the cellular nuclear import factors importin-α (karyopherin-α) and importin-β (karyopherin-β) [65-72] (‘Nuclear import’ in Figure 1). The M1 protein, after dissociating from vRNP complexes in late endosomes, is imported into the nucleus separately [73].

#### Virus replication and transcription

The replication and transcription of IAV genomic RNAs takes place in the nucleus and is catalysed by the trimeric viral polymerase complex composed of PB2, PB1, and PA subunits (‘Replication’ , and ‘Transcription’ in Figure 1). Viral RNA replication starts with the synthesis of a positive-sense copy of the vRNA, termed complementary RNA (cRNA) (reviewed in [74]). This cRNA is then copied to produce large amounts of vRNA (reviewed in [75,76]). Several host factors have been identified that may play a role in viral genome replication (reviewed in [77-79]).

Viral RNA transcription is initiated by the binding of PB2 to the 5′-cap structure of host mRNAs [80-82]. The endonuclease activity of PA [83] then ‘snatches’ the cap structure and the 10–13 nucleotides included with the cap serve as a primer for viral mRNA synthesis. The synthesis of viral mRNAs is carried out by the polymerase activity of PB1 [84]. The nuclear export of viral mRNAs is reviewed in York and Fodor [79]. Transcription proceeds until the polymerase complex stalls at a polyadenylation signal near the end of the viral RNA [85-88].

Two IAV mRNAs (derived from the two smallest vRNA segments, M and NS) are spliced to yield the M1 and M2, or the interferon antagonist (NS1) and nuclear export (NEP/NS2) proteins. Splicing is carried out by the host cell splicing machinery, but is likely regulated by NS1 [89,90], which binds to several cellular splicing components such as U6 small nuclear RNAs [91,92] and UAP56, a splicing factor involved in spliceosome formation [93,94].

#### Translation

Influenza viral mRNAs are translated by the host cell translation machinery (‘Translation’ in Figure 1); thus not surprisingly, several cellular translation factors such as eIF4A (eukaryotic initiation factor-4A), eIF4E, and eIF4G interact with viral mRNAs and/or polymerase complexes [95-98]. Upon IAV infection, host cell protein synthesis is limited, and IAV mRNAs are preferentially translated [99-101]. In particular, ‘cap-snatching’ may deplete newly synthesized, nuclear mRNAs of their cap structures, resulting in their rapid degradation before nuclear export and translation. In addition, the interaction of NS1 with the cellular PABII (poly(A)-binding protein II) [95,98] and CPSF (cleavage and polyadenylation specificity factor) proteins [102,103], and the interaction of the viral polymerase complex with the C-terminal domain of the largest subunit of cellular DNA-dependant RNA polymerase II (Pol II) [104,105] may contribute to the inhibition of host mRNA synthesis (reviewed in [106]).

After their synthesis in the cytoplasm, the viral polymerase subunit proteins and NP are imported into the nucleus via their nuclear localization signals [71,74,107-118] to catalyse the replication and transcription of vRNA. In addition, the M1 [64,119], NEP/NS2 [120], and NS1 [121] proteins are imported into the nucleus to execute their roles in vRNP nuclear export (M1 and NEP/NS2) or the processing and export of cellular and viral mRNAs (NS1) (reviewed in [122]).

#### vRNP export

The nuclear export of newly synthesized vRNP complexes requires the viral NEP/NS2 [123-126] and M1 [66,127,128] proteins. The latter is thought to form a bridge between vRNPs and NEP/NS2 [129-131], and M1 association with vRNP may require M1 SUMOylation [132]. In the nucleus, vRNPs destined for export are targeted to chromatin where they associate with Rcc1, and export is mediated by the cellular export factor Crm1 (‘vRNP export’ in Figure 1) [125,127,133] in a manner that is likely regulated by phosphorylation [65,128,134-137]. The cellular Y box binding protein 1 (YB-1) protein also associates with vRNPs in the nucleus, is likely exported from the nucleus in conjunction with vRNPs, and facilitates vRNP association with microtubules for transport to the plasma membrane (see below) [138].

Following their synthesis by the cellular translation machinery, the viral HA, neuraminidase (NA), and M2 proteins enter the endoplasmic reticulum (ER) where they are glycosylated (HA and NA) (reviewed in [139,140]) or palmitoylated (HA and M2). Cleavage of the HA proteins of highly pathogenic avian H5 and H7 viruses (which possess multiple basic amino acids at the HA cleavage site) into the HA1 and HA2 subunits occurs most likely by cellular furin-like proteases [141] in the *trans*-Golgi network; this cleavage event is critical for the virulence of influenza viruses [142,143].

#### Transport of virus proteins to the cell membrane

Transport of viral proteins to the plasma membrane (‘Transport to membrane’ in Figure 1) likely requires MTOCs (microtubule-organizing centers) [144,145], microtubules [144-146], and additional host factors including COPI (coatomer I) protein family members [147], a Rab GTPase (Rab11A) [145,148-150], and the HIV Rev-binding protein (HRB) [151].

#### Packaging and budding

At the plasma membrane, HA and NA associate with lipid rafts (membrane regions rich in sphingolipids and cholesterol) that are the site of influenza virus budding [152-160] (‘Packaging’ and ‘Budding’ in Figure 1). The assembly and virion incorporation of the eight vRNPs requires segment-specific packaging signals in the viral RNAs [161,162]. The M1 protein may play a role in the assembly process since it interacts with lipid membranes [163-165], vRNPs [130,131,166] (reviewed in [167,168]), and NEP/NS2 [129,169]. In addition, some evidence suggests the possibility that the M2 cytoplasmic tail mediates vRNP incorporation into the assembling virus particle [170].

Influenza virus budding does not require the proteins of the endosomal sorting complexes that are required to transport ESCRT complexes, which are utilized by a number of other viruses for budding. Rather, M2, which is found in the raft periphery [152,157,171], appears to mediate membrane scission and particle release [172]. This process may also require the cellular F1Fo ATPase [25]. The enzymatic activity of the viral NA protein removes sialic acids from host cells and from glycoproteins on virions, allowing virus release and preventing virion aggregation (reviewed in [55,75]).

#### Post-translational processing

Several post-translational modifications have been described for IAV proteins, including the glycosylation of HA (reviewed in [75,142]) and NA [173], the palmitoylation (S-acylation) of HA and M2 (reviewed in [174]), and the SUMOylation (i.e., conjugation with the *s*mall *u*biquitin-like *m*odifier) of M1 [132,175], NS1 [176,177], NP [175], PB1 [175], and NEP/NS2 [175] (‘Post-translational processing’ in Figure 1). Moreover, phosphorylation of M1 [137,178] and NP [107,179-183] may affect vRNP nuclear import and export [66,113,128,134]. Phosphorylation of NS1 [184] and PB1-F2 (a short protein synthesized from the PB1 gene; see below) affects virulence [185], although the mechanisms are not yet fully understood. These phosphorylation events are catalysed by several cellular kinases such as PKC (protein kinase C) which phosphorylates M1 [136], PB1-F2 [185], NS1 [184,186], and PB1 [186], or by CDKs (cyclin-dependent kinases) and ERKs (extracellular signal-regulated kinases), which phosphorylate NS1 [187].

#### Host responses

IAV infections trigger multiple host antiviral responses (reviewed in [188,189]). These interactions are summarized in the FluMap (Figure 1) and in the flowchart that depicts the different stages of the viral life cycle (Figure 3).

As a major host defence mechanism, pattern recognition receptors (PRRs) recognize infecting agents and trigger cellular antiviral responses (reviewed in [190]). To date, three major classes of PRRs [Toll-like receptors (TLRs); RIG-I-like receptors; NOD-like receptors (NLRs)] are recognized, all of which play a role in the defence against IAV infections. The activation of PRRs leads to increased production of type I interferon (IFN) and chemokines/cytokines, resulting in the upregulation of antiviral factors.

IAV infections are recognized by TLR3 [191,192], which acts through the adaptor molecule TRIF (TIR-domain-containing adapter-interferon-beta) to stimulate IFN-regulated factor 3 and NFκB (nuclear factor-kappa beta); TLR7 [193,194], which signals through the adaptor protein MYD88 (myeloid differentiation factor 88) and induces IRF7 (interferon regulatory factor 7) and NFκB; and RIG-I [195-198], which signals through MAVS (mitochondrial antiviral signalling), also known as IPS-1, and leads to the stimulation of IRF3, IRF7, and NFκB. Moreover, IAV infection activates the inflammasome [199-203], resulting in the cleavage and activation of pro-caspase-1, interleukin-1 beta (IL-1β), and IL-18.

PRR stimulation leads to the synthesis of IFNα/β, which binds to the ubiquitously expressed IFNα/β (IFNAR) receptor, resulting in the upregulation of the JAK/STAT (janus kinase/signal transducer and activator of transcription) pathway. JAK/STAT signalling induces the formation of a transcription factor complex (composed of STAT1, STAT2, and IRF-9) that upregulates the expression of IFN-stimulated genes (ISGs). A number of ISGs encode proteins with antiviral functions, such as PKR (protein kinase R), OAS (2′-5′-oligoadenylate synthetase), RNaseL (ribonuclease L), Mx, ISG15, IFITM family members, and viperin (see below for details). IAVs have thus evolved mechanisms to counter these host anti-viral defence strategies, primarily through the actions of the NS1 and PB1-F2 proteins.

NS1 is the major viral IFN antagonist ([204]; reviewed in [189,205]). It blocks RIG-I-mediated innate immune responses by targeting RIG-I [195,206] and/or TRIM25 (tripartite motif-containing protein 25) [207], and interferes with caspase-1 activation [208].

NS1 also interferes with the effects of several antiviral host factors. IAV infection activates PKR, resulting in the phosphorylation of the eukaryotic translation initiation factor eIF2α and the subsequent shutdown of protein synthesis. This activation is inhibited by NS1 [209-214]. NS1 also controls the antiviral activity of OAS and RNaseL, a cellular nuclease that degrades viral RNA [215]. ISG15 (interferon-stimulated gene 15) is an IFNα/β-induced, ubiquitin-like protein that conjugates to a wide array of cellular proteins, thus blocking their function. It affects IAV infection by interfering with the function of NS1 [216,217].

IAV infection stimulates the phosphoinsitide-3-kinase PI3K/Akt pathway [218-226], which has pro- and anti-viral functions (reviewed in [219]). In particular, this pathway is activated by NS1 binding to the p85 subunit of PI3K [218,221,224,226-228] and by IAV vRNAs via RIG-I [229]. Activation of the PI3K/Akt pathway is critical for efficient IAV replication [219,220], likely by preventing premature apoptosis [222,227,230-232].

The C-terminal four amino acids of most NS1 proteins comprise a PDZ ligand domain motif [233] that affects virulence [234-236] (reviewed in [237]), most likely through interaction with the cellular PDZ domain proteins Scribble, Dlg1 (disks large homolog 1), and membrane-associated guanylate kinase MAGI-1, -2, and −3 [238-240], which play roles in the regulation of apoptosis or tight junction formation.

NS1 also reduces the levels of IFNα/β mRNA by interfering with mRNA splicing [90-92,241] and the polyadenylation and nuclear export of cellular pre-mRNAs [90,91,102,241-246].

PB1-F2 is a short protein of 87–90 amino acids encoded by the +1 reading frame of most, but not all, IAV PB1 genes. It localizes to the mitochondrial membrane [247-249] where it interacts with the mitochondrial membrane proteins ANT3 (adenine nucleotide translocator 3) and VDAC1 (voltage-dependent anion-selective channel 1) [250], resulting in membrane depolarization [251,252] and the induction of apoptosis [247,248,250]. However, a recent study suggested that the induction of apoptosis may not be the major function of PB1-F2 [253]. Rather, PB1-F2 may interfere with the function of MAVS (mitochondrial antiviral-signalling protein) [254], and the resulting inhibition of IFN induction may contribute to PB1-F2-conferred increases in pathogenicity, inflammation, and the frequency and severity of bacterial co-infections [255-259]. In addition, PB1-F2 binding to PB1 affects the intracellular localization of the polymerase protein and reduces polymerase activity, potentially affecting virulence [260].

Other host antiviral factors include the Mx proteins [261-263], which most likely interfere with viral replication [264-266]; members of the IFITM protein family, which interfere with IAV cell entry [1,62,267]; and viperin, which executes its antiviral activity by disrupting lipid rafts that are critical for IAV budding [268].

Other important host responses to IAV infection include the mitogen-activated protein kinase (MAPK) signalling pathways, which regulate multiple cellular events including cell cycle control, cell differentiation, and apoptosis. All four of the currently recognized MAPK pathways [extracellular signal-regulated kinases 1/2 (ERK1/2); c-jun-N-terminal kinase (JNK); p38; and ERK5] are activated upon IAV infection [135,269-276]. Some of these pathways may have both pro- and anti-viral functions [135,274,277-279].

#### Antiviral compounds

The FluMap also captures antiviral compounds that are directed against a viral factor or a host target that is critical for efficient viral replication (reviewed in [280-283]). See Additional file 9 for a summary table.

Currently, there are two types of FDA-approved anti-IAV compounds: M2 ion channel inhibitors (amantadine, rimantadine), and NA inhibitors (oseltamivir, zanamivir).

M2 ion channel inhibitors block the ion channel in the viral envelope formed by the viral M2 protein. They prevent the influx of hydrogen ions from the acidic late endosome into the interior of the virion, a process that is necessary for the release of vRNPs into the cytoplasm. However, these inhibitors are no longer recommended for use in humans because most circulating IAVs are resistant to these compounds [284].

The NA inhibitors oseltamivir and zanamivir are the only antivirals currently recommended worldwide for human use. Both compounds block the enzymatic activity of NA that is critical for efficient virus replication [285-288]. Resistance to NA inhibitors has been described but is not widespread among currently circulating IAVs (reviewed in [289]).

Several new antiviral compounds are in different stages of clinical development and/or have been approved for human use in some countries, including two new NA inhibitors, peramivir [290,291] and laninamivir [292], and a viral polymerase inhibitor, T-705 [293-295].

Other strategies include the development of compounds that interfere with virus replication (ribavirin) [296,297], NP function (nucleozin) [298-301], NS1 function (several candidates) [302-304], or HA function [chemical compounds such as arbidol [305] that block HA-mediated membrane fusion, or monoclonal antibodies (MABs) directed against HA]. In particular, the development of monoclonal antibodies that target conserved regions of the HA protein and interfere with HA-mediated receptor-binding or fusion has received increased attention [306-314].

Host factors that are crucial for efficient IAV replication but dispensable for cell viability may be interesting drug targets since they are less likely to acquire resistance to an antiviral compound compared with IAV proteins (reviewed in [281,283]). For example, the sialidase DAS181 (Fludase, NexBio), which cleaves sialic acids on human bronchial tissue and inhibits IAV infection [315-317], is currently in Phase II clinical trials in the U.S. [283]. Several other approaches that are in early stages of development include: (*i*) protease inhibitors that block cellular enzymes required for HA cleavage [318-320]; (*ii*) specific inhibitors of MAPKs, such as U0126 (a MAPK/ERK inhibitor), which blocks the nuclear export of vRNP complexes [135,321]; (*iii*) NFκB inhibitors such as acetylsalicylic acid (ASA; commonly known as aspirin) [322], although aspirin may have adverse effects in IAV-infected individuals [323,324]; and (*iv*) agonists of sphingosine-1-phosphate (S1P) receptors, such as AAL-R, which reduce lung pathology upon IAV infection, likely because of their effect on dendritic cell activation, T-cell responses, and cytokine levels [325,326].

### *In silico* prioritization of potential drug targets

A critical quest in infectious disease research is to identify and prioritize novel potential therapeutic targets. In our *in silico* analysis of FluMap, we exploited a specific aspect of the network called controllability to identify molecules that, when inhibited, increase the likelihood of deregulating the virus replication cycle. Controllability is the ability to drive a network from any initial state to any desired state in a finite amount of time given a suitable choice of inputs [327]. From a biological network perspective, controllability analyses identify key molecular entities and processes that when perturbed can drive a biological system from a disease state to a healthy state [328].

To begin, we identified the smallest set of driver nodes (molecules, complexes, etc.) needed to attain complete control of all of the other nodes in the network. The size of this smallest set was directly related to how difficult it was to control the network in question. Networks that demand a large set of driver nodes are inherently more difficult to control. Further, as nodes are removed from the network, the identity of the driver nodes may change but, more importantly to our application, the number of driver nodes – and the associated difficulty of controlling the network – may remain fixed or also change. Thus, the second step of the analysis involved identifying ‘critical’ nodes that when removed from the network, *increased* the number of driver nodes necessary to elicit complete control, that is, increase the difficulty in controlling the network [329]. From a therapeutic perspective, inhibition of critical nodes/links would make it increasingly difficult for the virus to maintain control of the replication process. Further, controllability analysis can also be performed for the network links. Lastly, we investigated whether the critical nodes/links are associated with more commonly used network topology measures (e.g., nodes with a high number of neighbours (degree) or nodes that are bottlenecks in the network (betweeness)).

**Figure 4:**
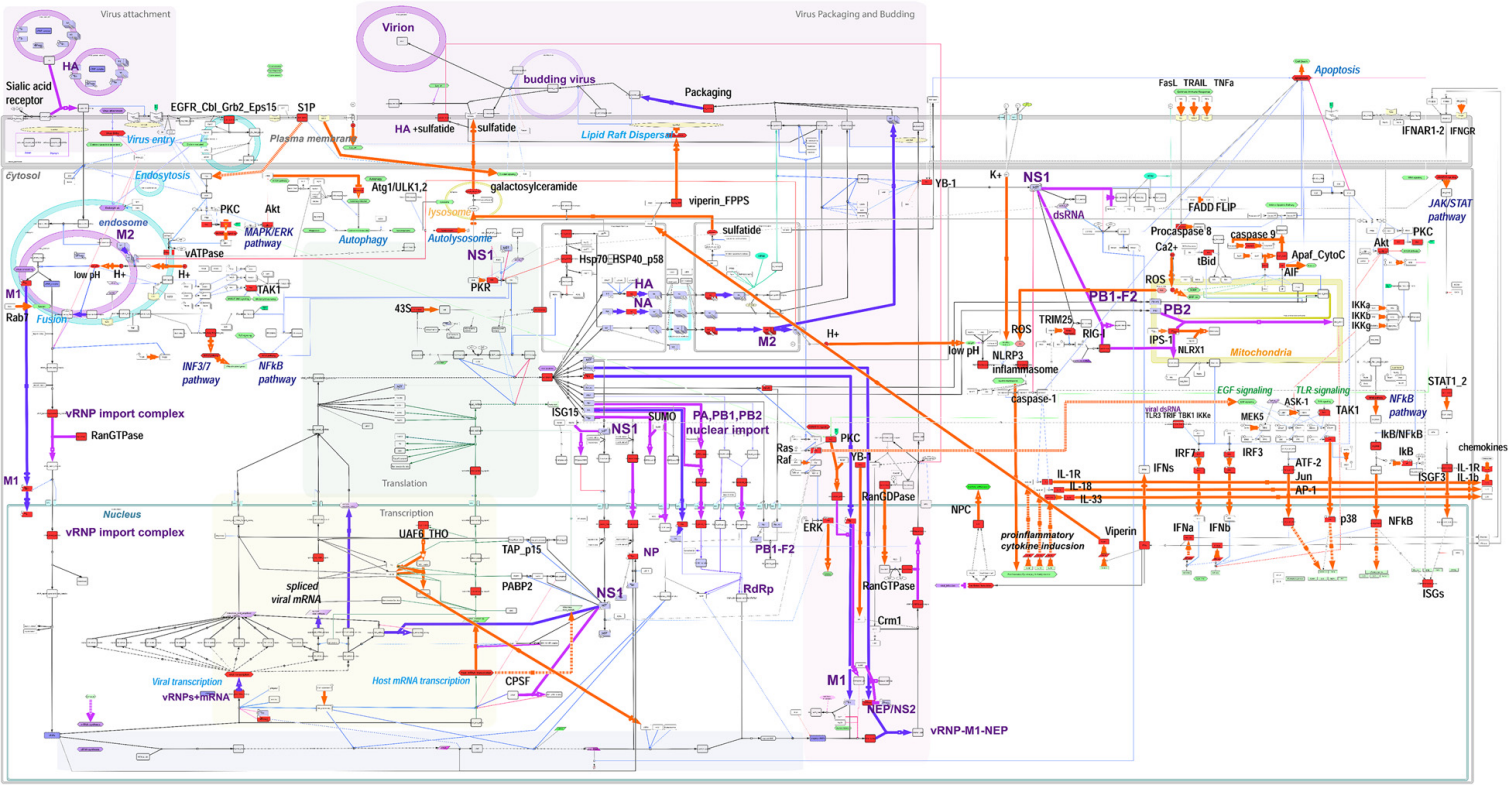
**Controllability analysis.** Critical factors (nodes) identified by the controllability analysis are shown in red. Thick magenta edges indicate critical interactions between viral and host proteins, purple edges indicate virus factor interactions/transitions (for example, transport processes), and orange edges indicate interactions between host factors.

To facilitate the above analyses, we converted FluMap to a binary network by taking the direction of connections while ignoring the type of reaction (catalysis, inhibition etc.) (Figure 4; Additional file 10 and Additional file 11). Note that controllability analysis does not use the type of reaction (e.g., catalysis, inhibition etc.). Thus, ignoring the type of reaction does not affect the results.

Within the FluMap, we found that 256 (41.2%) of the nodes were driver nodes and 112 (18.0%) were critical nodes. Among the 137 critical links (15.3%), ~15% accounted for interactions among viral factors, whereas ~10% accounted for virus-host interactions. The remaining two-thirds accounted for reactions between host factors. Compared with previous studies [327], the driver nodes ratio of the FluMap is similar to that of metabolic networks (30%–40%), and lower than the gene regulatory networks (>80%).

Topology analysis revealed that critical nodes tended to have a higher degree and higher betweenness than noncritical nodes (two-sided Wilcoxon rank sum test [WRST] of the degree and log_10_ of the betweenness; P < 2.2E-16 and P = 3.452e-06, respectively, see Additional file 12). By using the node degree to prioritize the critical nodes, we found that the nuclear pore complex (NPC) and the three host proteins, Akt, PKC, and the Ran/GTPase complex (which plays a critical role in the export of proteins from the nucleus to the cytoplasm), are both critical and highly connected within the network. PKR and Y-box binding protein 1 (YB-1) come in the second tier. YB-1 is reported to assist in the transport of influenza virus RNP to microtubules [138]. Perturbation of these complexes/factors would thus be expected to have the greatest impact on the IAV life cycle.

Among the *137 critical interactions* identified, we did not find that critical interactions have a higher or lower edge betweeness than noncritical interactions (P=0.1, WRST of the log_10_ of the edge betweenness), but we did find that the ISG15-NS1 interaction and several interactions related to pH control involved molecules with high degree. Our controllability analysis identified several current antiviral compounds and targets, such as M2 ion channel inhibitors (which affect the pH inside the virion), the targets of sialidase, and viral polymerase inhibitors (Figure 4).

Our results suggest that the controllability analysis, together with network topology characteristics, can identify important factors for the viral life cycle that may be potential therapeutic targets as well as known drug targets. Given that the current map is constructed by manual curation, many important edges and nodes may be missing, so that the robustness of the controllability analysis cannot be assessed. Nonetheless, we show the potential of identifying and prioritizing critical nodes and edges that may be targeted for antiviral drug development.

## Utility and discussion

Here, we present FluMap, a comprehensive pathway map for IAV infections. This map is the most recent version of the IAV host-virus interaction map and includes a significantly higher number of factors than previous versions. It is intended to provide a platform for data sharing, community curation, and *in silico* analysis, such as network controllability analysis. We have made FluMap accessible online to allow for pathway and annotation browsing. We have also provided interactive features that will allow the research community to actively participate in improving and updating FluMap.

### FluMap as a data analytic platform

We applied a network controllability analysis to demonstrate that maps like FluMap can be used for *in silico* analysis. Although the controllability analysis we applied here does not take into consideration the nature of the interaction (for example, activating or inhibitory), our analysis identified several events known to be critical for the IAV life cycle, suggesting that the algorithm [327] can be effectively applied to process-descriptive pathway networks such as FluMap to identify and prioritize factors that could be targeted to affect the IAV life cycle. In addition to known targets, our analysis also identified factors that are not currently recognized as critical, such as YB-1; further experimental testing could address the significance of these events in IAV infections.

A comprehensive map such as FluMap can also be used to analyze large-scale data sets (obtained from ‘omics’ or siRNA inhibition studies) by using the data mapping function of CellDesigner or other visualization tools.

For a deeper insight into IAV virus-host interactions, the next step in pathway modeling is the integration of additional datasets of host responses to IAV infections. FluMap includes critical host response factors such as RIG-I, PKR, and the NLRP3-inflammasome. However, the pathways regulated by these factors are complex and a significant amount of ‘cross-talk’ occurs between the pathways, making it extremely challenging to comprehensively map host responses. Here, the integration of additional experimental data as they become available will improve our understanding of host responses to IAV infections. Moreover, future versions of FluMap could integrate intercellular reactions, such as events stimulated by interferons and cytokines/chemokines.

Lastly, a key distinction of FluMap compared with previous influenza replication cycles is the inclusions of strain-specific information. There are strong differences between the pathogenic potential of individual virus strains, and highly pathogenic strains may exploit different host machinery to ensure rapid replication and immune suppression [330-333]. Within FluMap, users can exploit the various annotations tools to analyse isolate-specific pathway interactions and attempt to identify critical molecular events associated with highly pathogenic infections. As future studies with H5N1, H7N9, or reconstructed Spanish influenza viruses reveal more information regarding virus-host interactions, the FluMap presented here will provide a basis for rapid consolidation and *in silico* exploration.

## Conclusions

We constructed a publicly available knowledge base called “FluMap” that contains 960 factors and 456 reactions. All reactions are annotated with PubMed IDs in the Notes section and isolate-specific information is available from many interactions; the entire map is annotated with 476 papers. FluMap is a comprehensive Influenza A virus replication life cycle and host response map, and is expected to be a valuable guidance map for those who study influenza infection.

## Availability and requirements

The FluMap is accessible at http://www.influenza-x.org/flumap/.

## Abbreviations

IAV: Influenza A virus; SBML: Systems biology markup language; SBGN: Systems biology graphical notation.

## Competing interests

The authors declare that they have no competing interests.

## Authors’ contributions

YM, HK, and YK conceived the idea of FluMap. YM, HM, and MK developed the map. AJE, TW, SW, SF, GN, and TL reviewed and curated the map. TH, SG, JS, and YM conducted the controllability analysis. YM, HM, and GN wrote the manuscript. All authors approved the manuscript.

## Supplementary Material

Additional file 1 **FluMap building and workflow of literature-based pathway modeling.** (a) FluMap was built based on information from the literature and from several pathway databases such as Reactome, KEGG, and PANTHER. The resulting map captures the viral life cycle and host responses. Extensive annotations are provided. We then manually generated a simplified map for high-level overview, and a map in which arrows outline the sequence of events during IAV infection (i.e., binding, internalization, nuclear import, etc.). We conducted controllability and network analyses over the FluMap to identify nodes essential to the replication process. Key interactions and nodes from these analyses are highlighted. (b) Summary of the literature-based pathway modeling process that converts and integrates textual information into a graphical representation. FluMap allows the community to browse, use, and comment on the information provided; this interface with the research community is shown in green. Click here for file

Additional file 2 **How to browse FluMap.** This document explains how to browse FluMap at the website http://www.influenza-x.org/flumap/, and shows its graphical notation scheme, as well as the annotation policy we adopted for curation of the map. It also describes how to open the map file with CellDesigner for further analysis or modification, and how to curate the map on the Payao system (http://www.payaologue.org). Click here for file

Additional file 3 A poster version of FluMap. Click here for file

Additional file 4 **SBML map file of FluMap.** The SBML map file FluMap.xml can be browsed using CellDesigner. Please download CellDesigner at http://www.celldesigner.org/, install it, and open the SBML file FluMap.xml to browse FluMap by using CellDesigner. For usage of the software, see the documentation provided at the CellDesigner website: http://www.celldesigner.org/documents.html Click here for file

Additional file 5 **Entities & Reactions List of FluMap.** This is a list of the entities (such as proteins, genes, etc.) and reactions (interactions between entities) in FluMap. Click here for file

Additional file 6 **Reference List of FluMap.** This contains all of the references annotated in FluMap. Click here for file

Additional file 7 **SBML map file of the simple version of FluMap.** The SBML map file of the simplified version of the IAV virus-host interaction map can be browsed by using CellDesigner. Please download CellDesigner at http://www.celldesigner.org/. For detail usage of the software, see the documentation provided at the CellDesigner website: http://www.celldesigner.org/documents.html Click here for file

Additional file 8 **SBML map file of the flowchart version of FluMap.** The SBML map file of the IAV virus-host interaction timeline can be browsed by using CellDesigner. Please download CellDesigner at http://www.celldesigner.org/. For detail usage of the software, see the documentation provided at the CellDesigner website: http://www.celldesigner.org/documents.html Click here for file

Additional file 9 **Antiviral Drug List.** This is a list of the influenza-related antiviral drugs. Click here for file

Additional file 10 **Controllability Analysis.** This document describes the protocol for the controllability analysis we conducted with FluMap. Click here for file

Additional file 11 **Controllability Analysis Results.** This file contains the results of the controllability analysis, listing the critical, ordinary, and redundant nodes/links. Click here for file

Additional file 12 **Topology Analysis Results.** This file contains the results of the topology analysis based on the controllability analysis results to prioritize the target candidates. Click here for file
