# Supplementary figures and images for: A comprehensive map of the influenza A virus replication cycle
Source: BMC Syst Biol. 2013 Oct 2;7:97. doi: 10.1186/1752-0509-7-97 (PMC3819658; doi:10.1186/1752-0509-7-97)

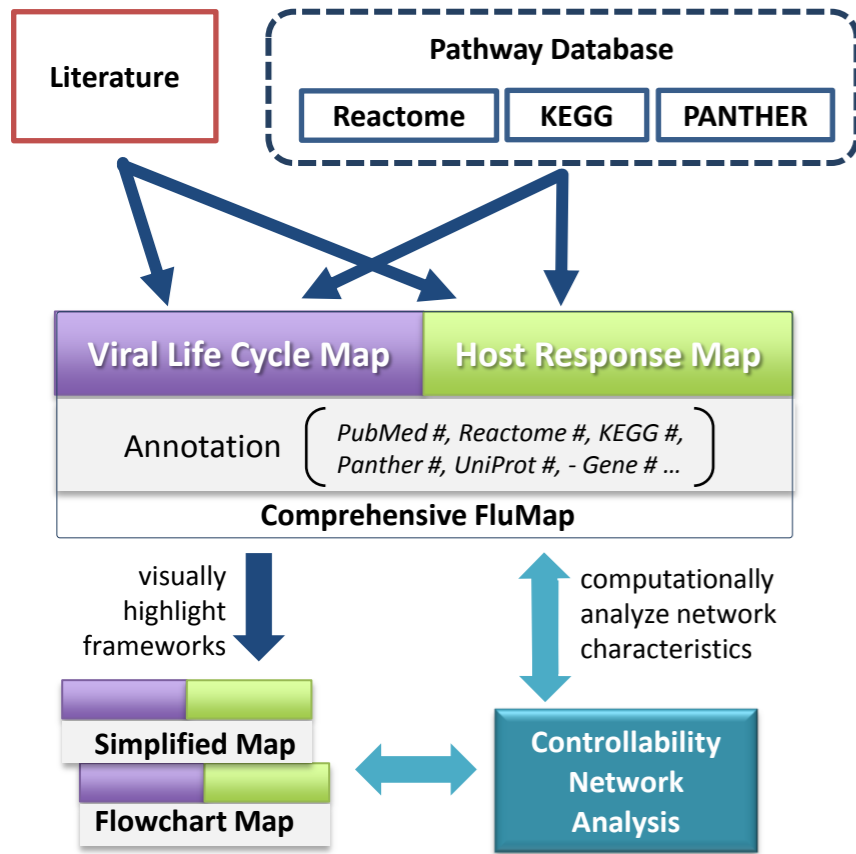

(a)

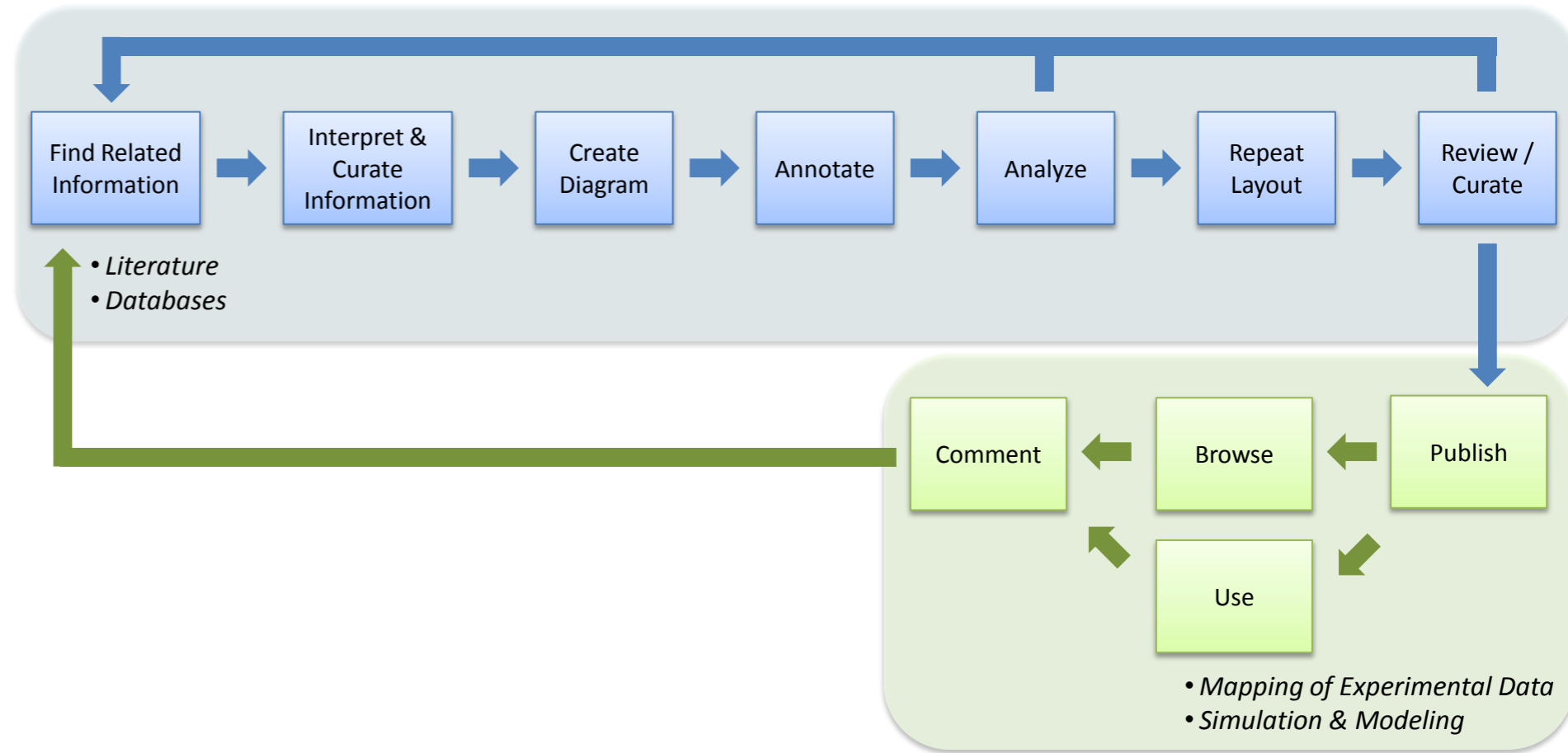

(b)

Supplement: Additional file 1 — FluMap building and workflow of literature-based pathway modeling. (a) FluMap was built based on information from the literature and from several pathway databases such as Reactome, KEGG, and PANTHER. The resulting map captures the viral life cycle and host responses. Extensive annotations are provided. We then manually generated a simplified map for high-level overview, and a map in which arrows outline the sequence of events during IAV infection (i.e., binding, internalization, nuclear import, etc.). We conducted controllability and network analyses over the FluMap to identify nodes essential to the replication process. Key interactions and nodes from these analyses are highlighted. (b) Summary of the literature-based pathway modeling process that converts and integrates textual information into a graphical representation. FluMap allows the community to browse, use, and comment on the information provided; this interface with the research community is shown in green. [file 1752-0509-7-97-S1.pdf]
