# Supplementary material for: A comprehensive map of the influenza A virus replication cycle
Source: BMC Syst Biol. 2013 Oct 2;7:97. doi: 10.1186/1752-0509-7-97 (PMC3819658; doi:10.1186/1752-0509-7-97)
Supplement: Additional file 2 — How to browse FluMap. This document explains how to browse FluMap at the website http://www.influenza-x.org/flumap/, and shows its graphical notation scheme, as well as the annotation policy we adopted for curation of the map. It also describes how to open the map file with CellDesigner for further analysis or modification, and how to curate the map on the Payao system (http://www.payaologue.org). [file 1752-0509-7-97-S2.pdf]

Additional file 2: How to browse the FluMap.

A) How to browse the FluMap on the website
B) Graphical Notation of the FluMap
C) Notation Example of the FluMap
D) Annotation Policy of the FluMap
E) How to browse the Map on CellDesigner.
F) How to browse the Map on Payao

A) How to browse the FluMap on the website

Open http://www.influenza-x.org/flumap/home.html

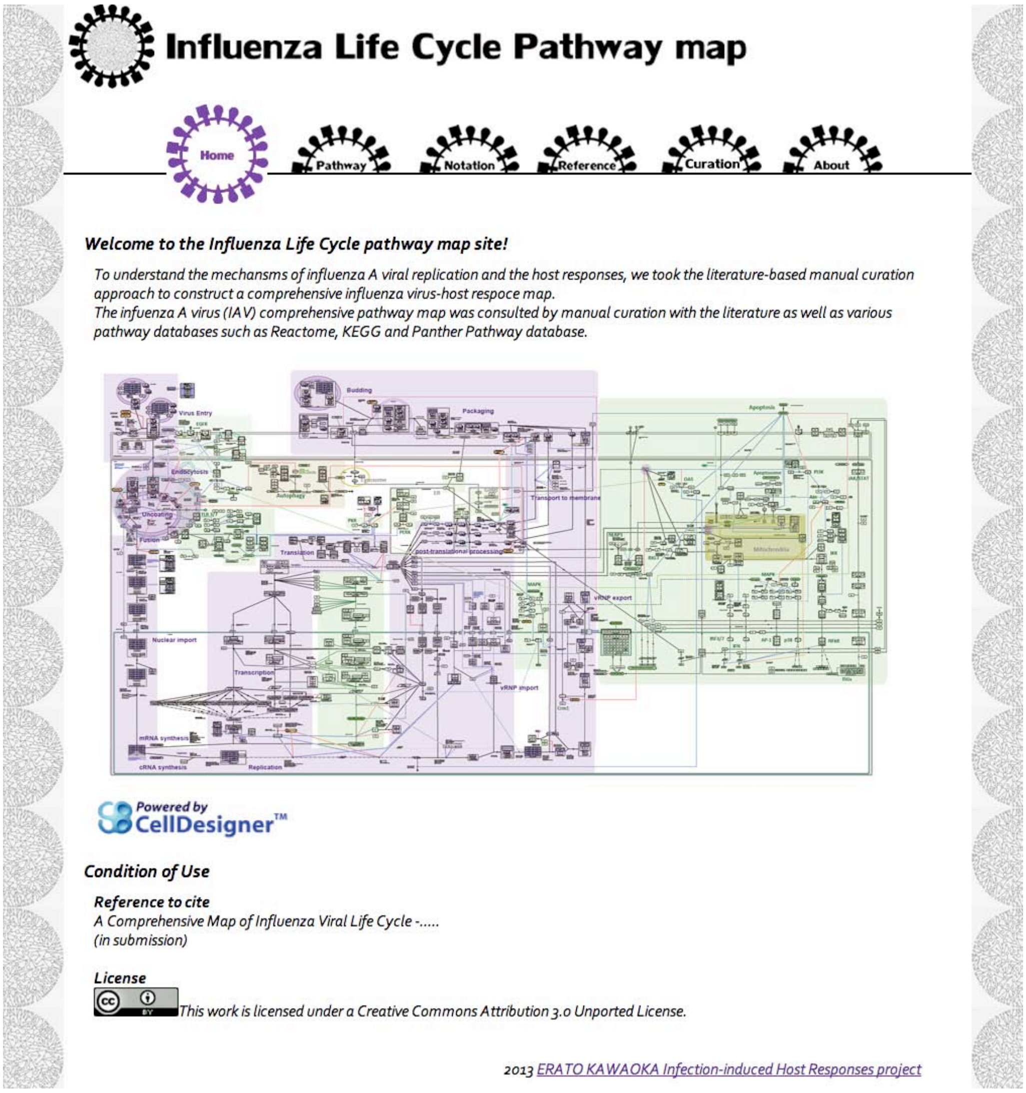

1. Click the [Pathway] tab
   The FluMap pathway page is displayed.
   The maps are embedded like a "Google Map".

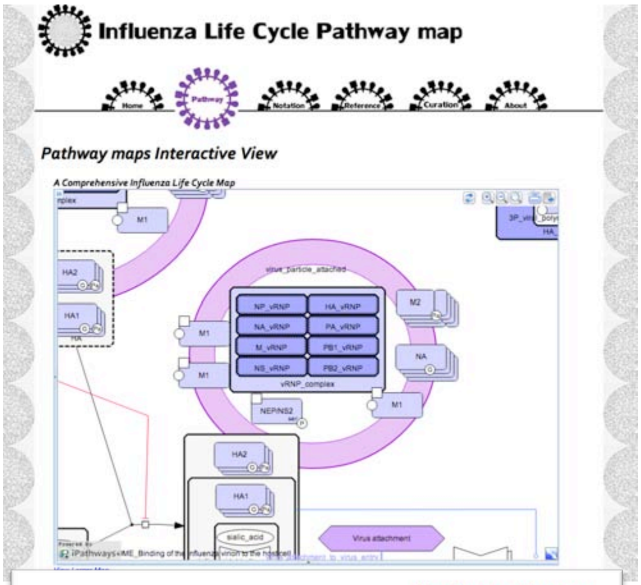

2. Navigate the map using the navigation buttons in the upper right part of the screen.
   You can zoom in/out, zoom fit and refresh the map.
   You can also print/export the image.

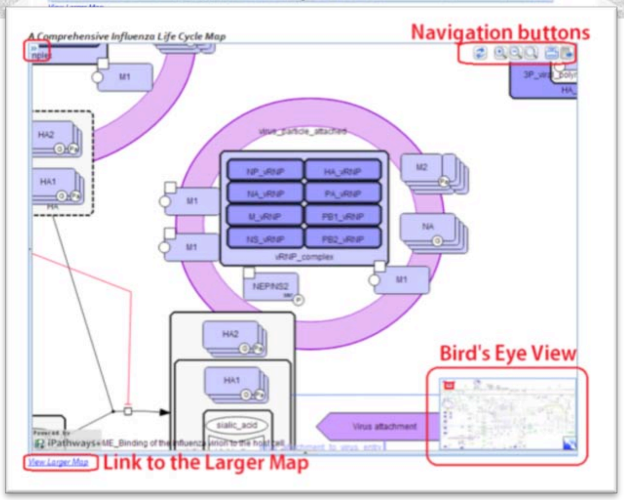

3. To display the Layer, click on the left top arrow button to expand the left panel menu.
   Then select the layer of the Notes, MIRIAM etc.

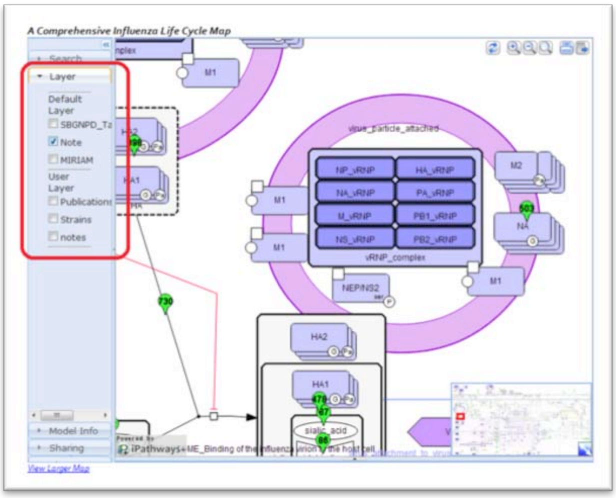

5. Click the "View larger Map" link

→ Go to the iPathways+ Website.

6. Click the [Layer] tab and check the [Note] checkbox.

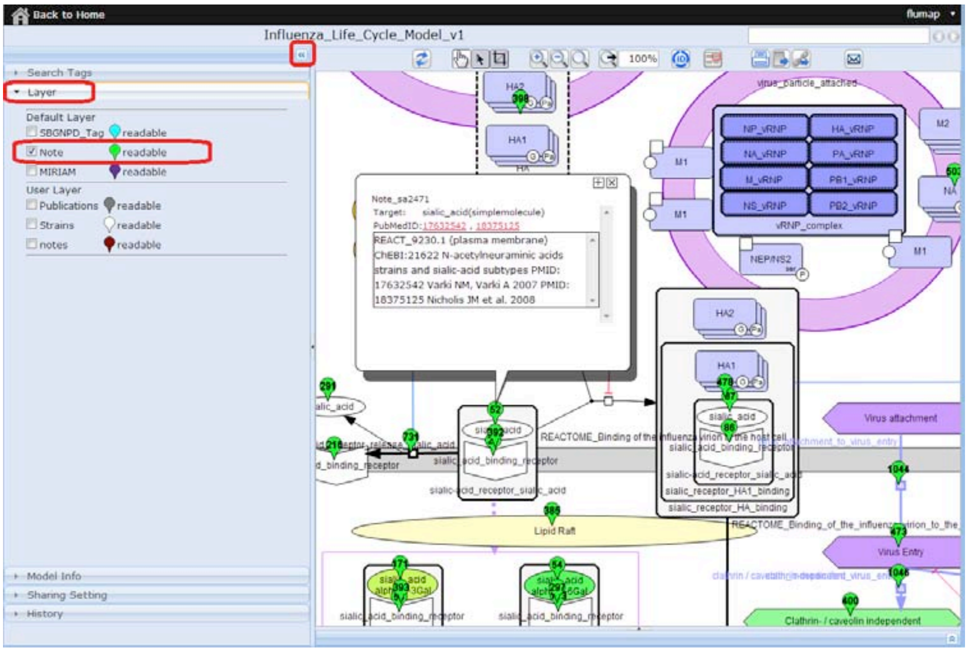

7. Click each number tag (e.g., “52”), and the PubMedID link
→ Go to the Pubmed website.

For further detailed operations, please refer to iPathways+ FAQ:
http://ipathwaysplus.unit.oist.jp/ipp/faq.php

B) Graphical Notation of the FluMap.

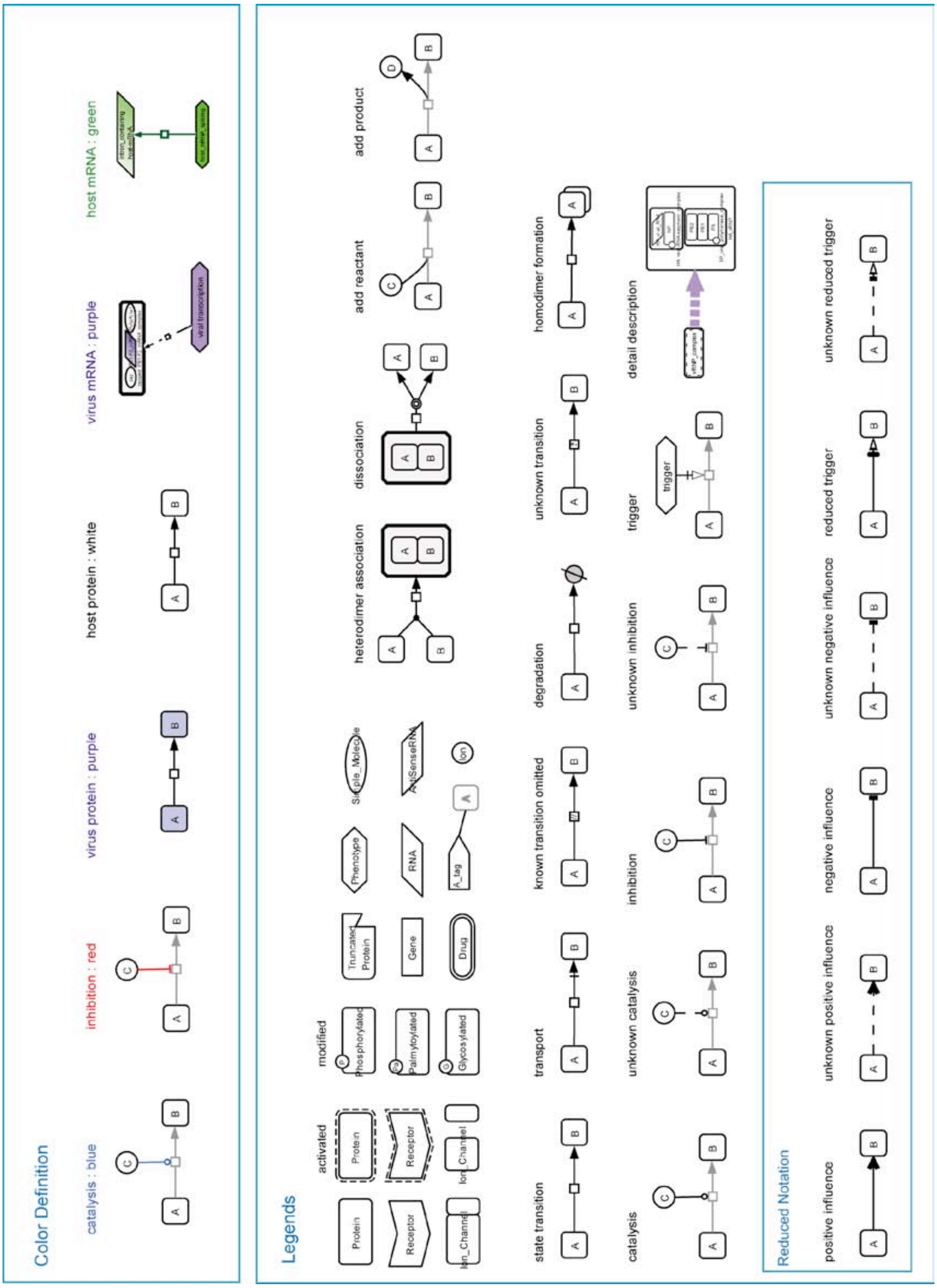

For details on the graphical notation of CellDesigner ver4.3, please refer to http://celldesigner.org/help/CDH_Component_T.html

C) Notation Example of the FluMap

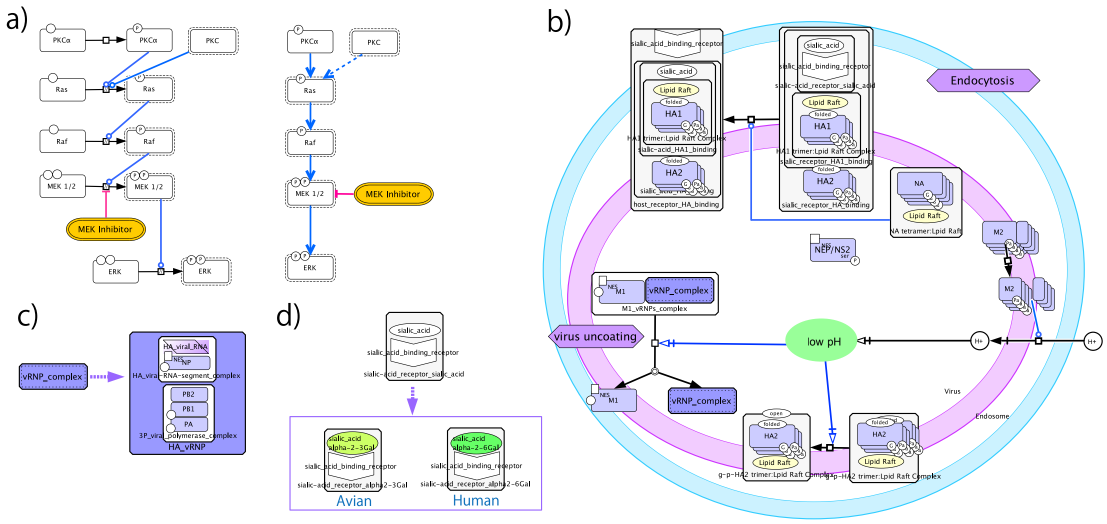

(a) Examples of 'process description diagram' and 'simplified notation' (which is called "reduced notation" in CellDesigner 4.3). (b) An example of a reaction that is triggered by a change of intracellular parameters. The low pH in late endosomes that triggers fusion of viral and endosomal membranes is shown in light green. (c) Complexes such as the Influenza A virus (IAV) polymerase complex (composed of the IAV PB2, PB1, and PA proteins) can be shown in a detailed ('complex') or condensed ('compact') view. To see all of the subunits of the ribosome or proteasome, the SMBL file can be opened in CellDesigner and the detailed 'complex' mode of the selected components can be toggled to the 'normal' or 'compact' mode. (d) Strain-specific events, such as sialic acid preferences of human and avian influenza viruses, are overlaid as a 'layer annotation'.

D) Annotation Policy of the FluMap (Full)

	Reaction		Species		Protein/ Gene/RNA	Layer
	Notes	MIRIAM	Notes	MIRIAM	Notes	
PubMed	◎	◎	○	○		◎
ReactomeID	○	○	○	○		
Panther/KEGGID	○					
UniProtID			○		◎	
GeneID			○		○	
Category	◎					
Location			○			
Memo	○		○		○	○

◎: required ○: optional

E) How to browse the Map on CellDesigner

1. Download and install **CellDesigner Ver4.3** or higher (available from "http://celldesigner.org").
2. Open the map file from the [File]-[Open] menu.

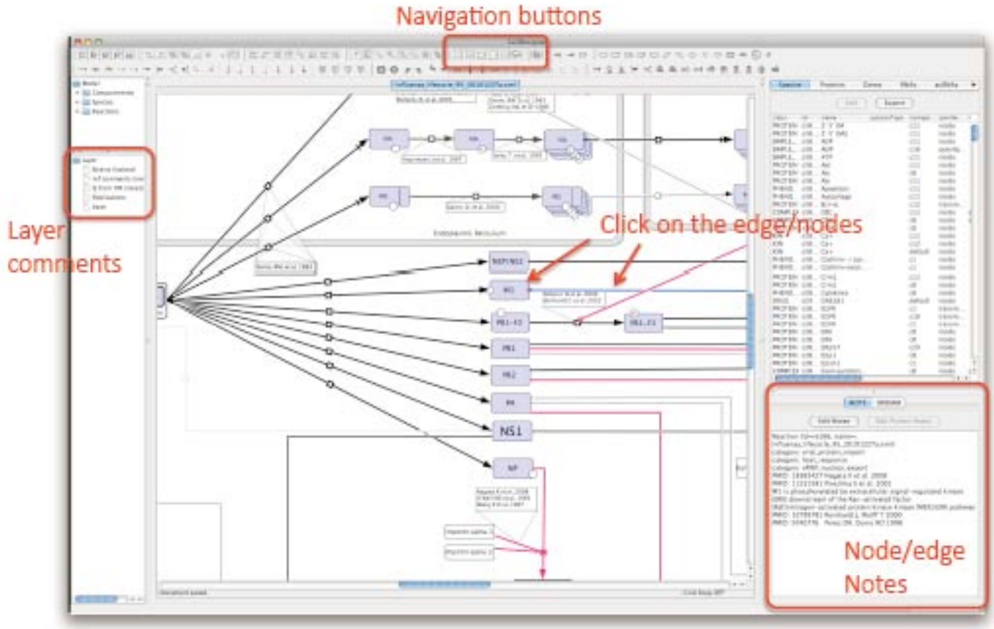

The xml files are available from Additional files 4, 7, and 8.

3. To find the annotations, click on the edges/nodes on the main canvas, then check in the Notes / MIRIAM section.
4. To browse the relevant information of the IDs (i.e., PubMed, GeneID, UniProtID) described in the Notes section, click on the Species/Reactions of your choice and select [Database]-[Connect to PubMed], [Connect to Entrez Gene] or [Connect to UniProtID].
5. To browse the relevant links described in the MIRIAM section, click on the item in the MIRIAM's table, then press the [Access] button.

For further detailed operations, please refer to the help page or documents at: http://www.celldesigner.org/index.html

F) How to access the Map on the Payao system

http://www.payaologue.org/

1. Click the [Open PAYAO] button.

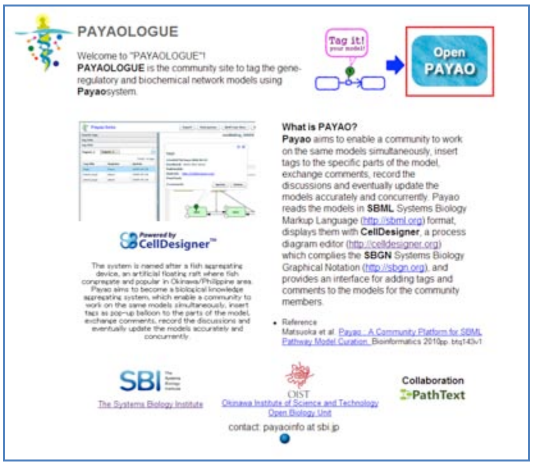

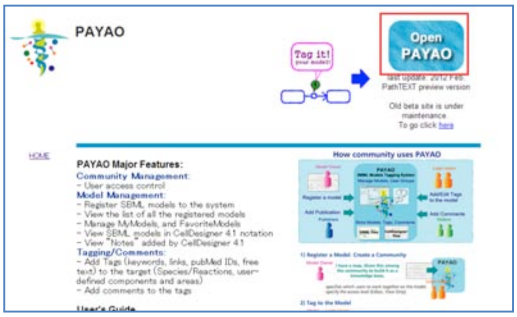

2. Login to the Payao system: Input "User name" and "Password"

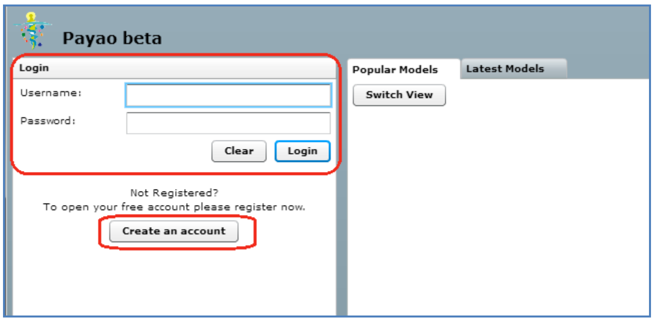

If you do not have an account, register by using the [Create an account] button.

3. Click the [View] button of the map that you want to see.
(You can also search the map in the [Search Models] tab)

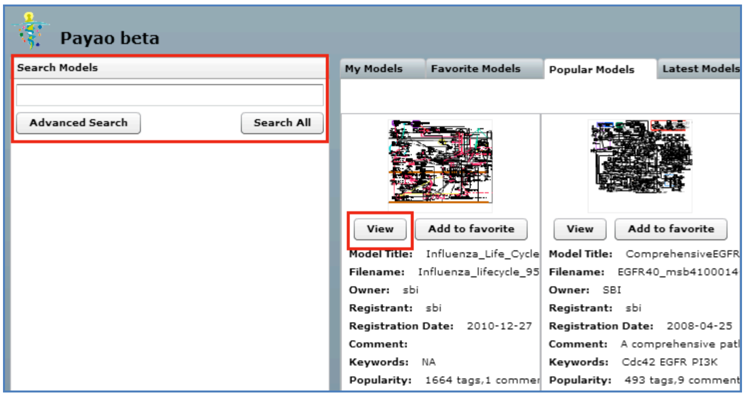

4. Click the balloon on the map to check the tag comments.
   You can see the Notes/MIRIAM information stored by CellDesigner, or the comments added by Payao users.

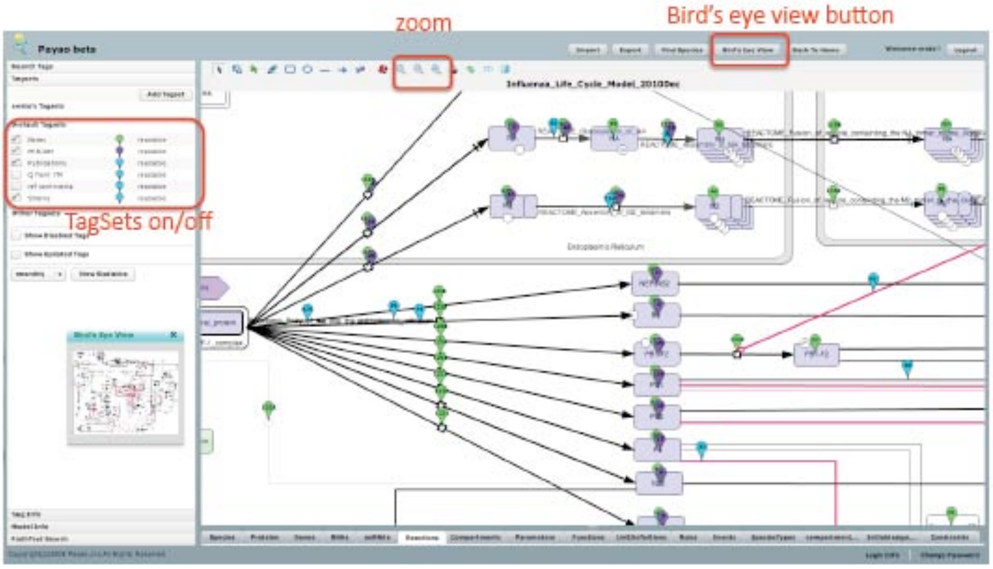

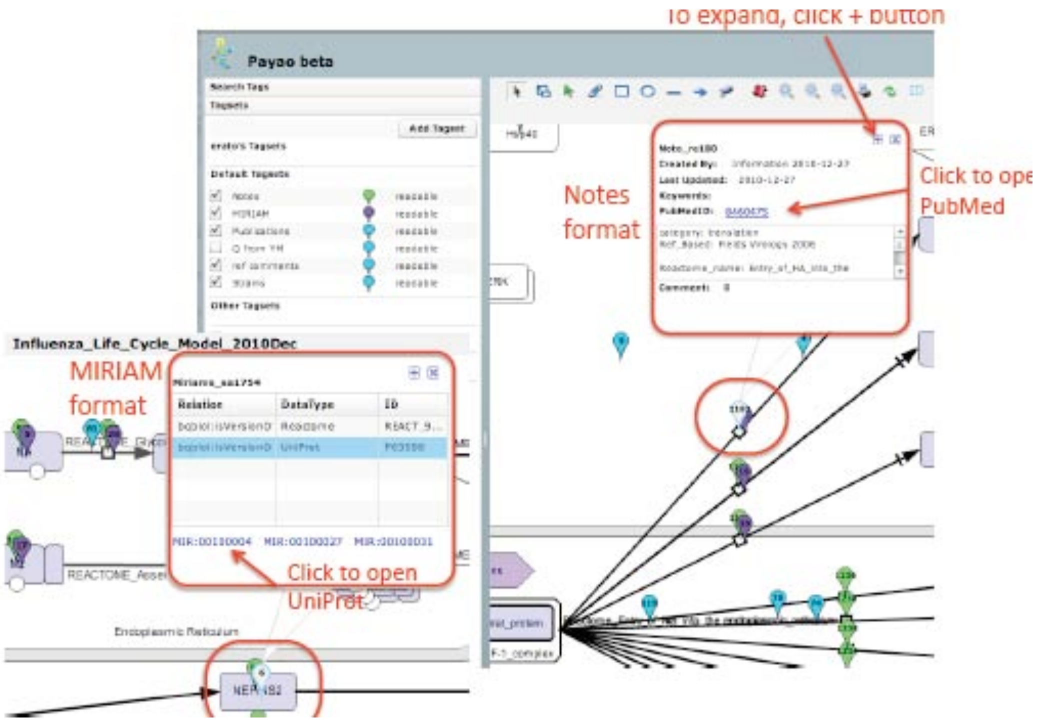

5. You can add new Tags or Comments.

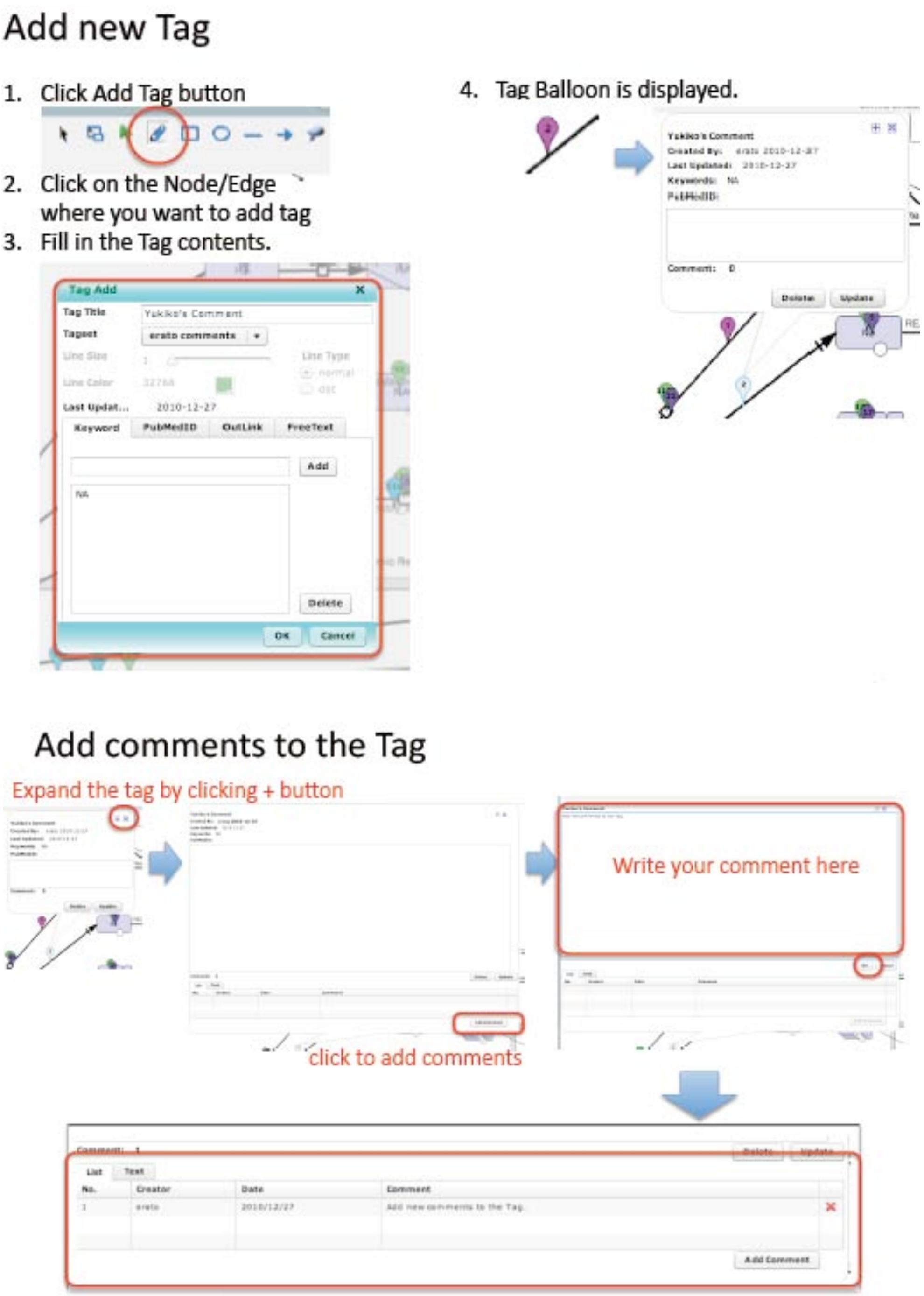

For further details, please refer to the PAYAO Users Guide:
http://payao.unit.oist.jp:8080/payaologue/doc/PAYAO_Users_GuideE11.pdf
