# Supplementary material for: A comprehensive map of the influenza A virus replication cycle
Source: BMC Syst Biol. 2013 Oct 2;7:97. doi: 10.1186/1752-0509-7-97 (PMC3819658; doi:10.1186/1752-0509-7-97)

Influenza A Virus Life Cycle Map Ver.1.0

ERATO KAWAOKA Infection-induced host responses Project, Japan Science and Technology Agency

produced by CellDesigner ver4.2 + Adobe Illustrator
CellDesigner is available at http://celldesigner.org/

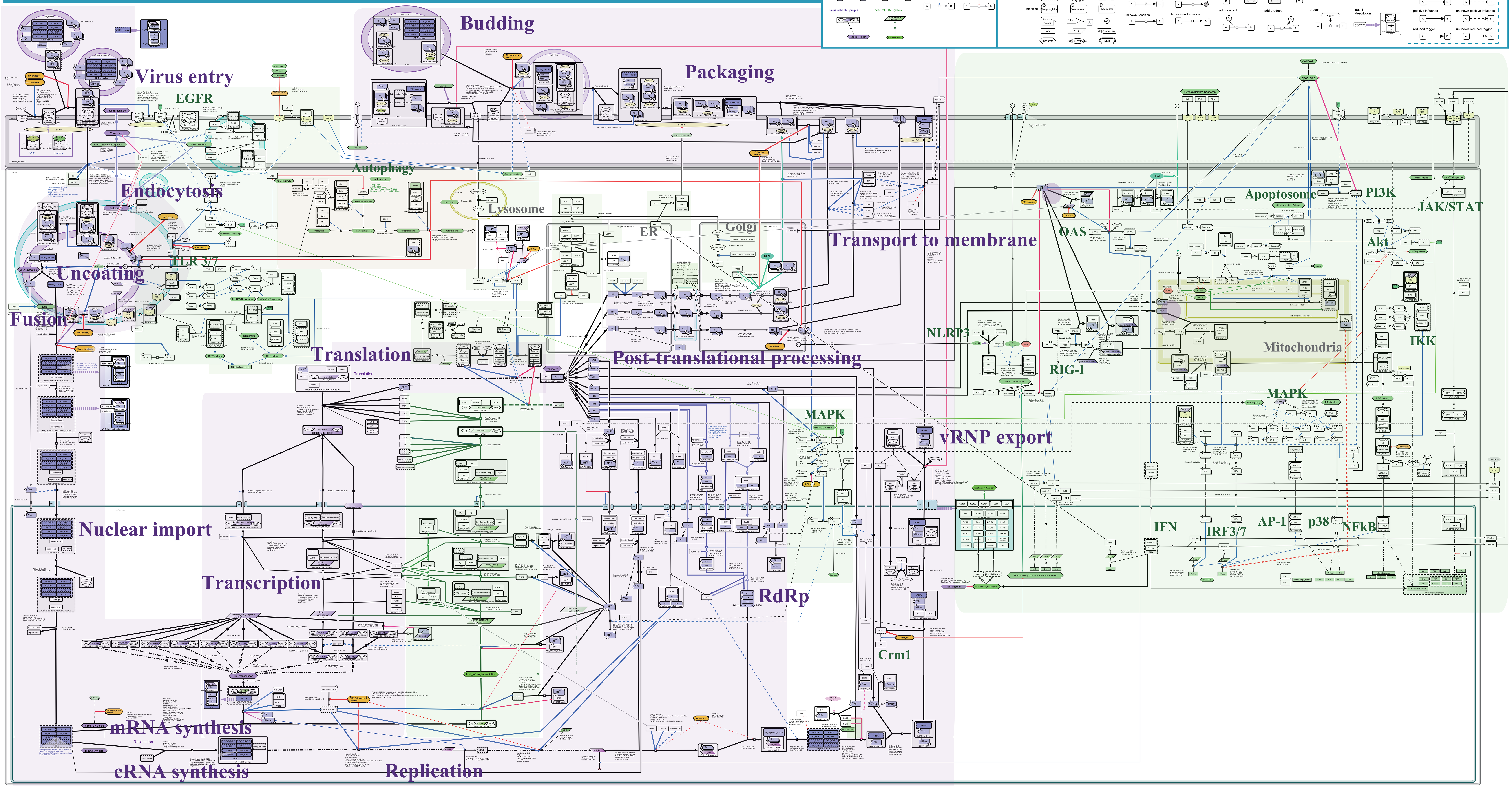

Supplement: Additional file 3 — A poster version of FluMap. [file 1752-0509-7-97-S3.pdf]
