# Supplementary material for: A comprehensive map of the influenza A virus replication cycle
Source: BMC Syst Biol. 2013 Oct 2;7:97. doi: 10.1186/1752-0509-7-97 (PMC3819658; doi:10.1186/1752-0509-7-97)
Supplement: Additional file 10 — Controllability Analysis. This document describes the protocol for the controllability analysis we conducted with FluMap. [file 1752-0509-7-97-S10.pdf]

Additional file 10: Controllability analysis

Methods

1) Preprocessing. We took the complete FluMap (Figure 1, Additional file 4) and deleted all of the drug nodes and their associated interactions.

2) Conversation. Controllability and network topology analysis can be applied to any “directed” or “undirected” network but such networks typically cannot describe the type of interaction (e.g., catalytic versus binding events) and they generally lack the degree of context that is described in well annotated, biochemical maps. Nonetheless, analyses of such abstracted network descriptions have proven valuable in gene essentiality (Jeong *et al.* 2001) and drug target identification studies (Hase *et al.* 2009).

In this work, we used Perl scripts to convert the FluMap to a simple bipartite graph in which binding events are described as follows: if A binds B to form complex C, then in the bipartite graph, both A and B are assigned edges to C that point toward C. Certain reaction types cannot be distinguished in graphs and result in the same style of linkages. Consider as an example if the reaction A->B is either catalyzed or inhibited by C. In a typical bipartite graph, catalytic and inhibitory events cannot be distinguished and for both cases, the connections would be A and C are connected to B with arrows pointing towards B.

3) Identify initial number of driver nodes. By using “maximum matching” in the bipartite digraph, we determined the minimum number of driver nodes that are required to fully control the FluMap. Liu *et al.* showed that the minimum set of driver nodes needed to gain full control of the network is determined by the “maximum matching” in the network; that is, the maximum set of links that do not have common start or end nodes (see (Liu *et al.,* 2011) for additional discussion). Thus, we used the Hopcroft-Karp algorithm (Hopcroft & Karp, 1973) to determine the driver nodes of the full FluMap. We found 256 driver nodes in the map (see Additional file 11).

4) Indentify critical links. A node (link) in a pathway is defined as “critical”, if the absence of the node (link), requires an increase in the number of driver nodes to fully control the pathway. In our case, if more than 256 driver nodes were needed to exact controllability when node *j* is removed, then node *j* is

critical. Thus, we iteratively stepped through the map, removing either a node or link at each step, and calculated the number of driver nodes needed for controllability. In all, we identified 112 critical nodes and137 critical links in the IAV map. These results are details in Additional file 11.

5) Illustrating results. We highlighted the critical nodes/links in the FluMap in by using color (See Figure 4).

6) Comparing critical nodes/links to network topology characteristics. In R, using the igraph package (Csardi & Nepusz, 2006), we determined the node degree and betweenness and the edge betweenness (See Additional file 12).

7) Node/link prioritization. We prioritized the critical nodes/links to filter the potential drug target candidates by using the network controllability and topology analysis results.

Table S10-1: Controllability Analysis Results of the Complete FluMap

Analysis item	Total
Number of driver nodes	256
Fraction of driver nodes	0.412238325
Total number of nodes	621
Number of critical nodes	112
Number of ordinary nodes	293
Number of redundant nodes	216
Fraction of critical nodes	0.180354267
Fraction of ordinary nodes	0.471819646
Fraction of redundant nodes	0.347826087
Total number of links	897
Number of critical links	137
Number of ordinary links	704
Number of redundant links	56
Fraction of critical links	0.152731327
Fraction of ordinary links	0.78483835
Fraction of redundant links	0.062430323

The controllability analysis results from the comprehensive FluMap are similar to those from the simplified FluMap (Figure 2, main text); there are no significant differences in the fraction of driver nodes, that is, of critical, ordinary, and redundant nodes/links, between the comprehensive and simplified maps. For simplicity, the results from the simplified FluMap are not shown.

Note: Additional file 11 shows the lists of reaction IDs associated with critical, redundant, and ordinary links and some reaction IDs that share only one link. In the lists, there are 138 reaction IDs associated with 137 critical links, that is, two reaction IDs (re311 and re722) associate with a critical link from nodes s381084 to s384737, whereas the remaining 136 reactions associate with 136 critical links. Similarly, 725 reactions associate with 704 ordinary links.

See also:

- **Additional file 11:** The lists of critical nodes/links and driver nodes.
- **Additional file 12:** The topology analysis results.

References

- Csardi G, Nepusz T (2006) The igraph software package for complex network research. *InterJournal* **Complex Systems:** 1695
- Hopcroft J, Karp R (1973) An n^5/2 Algorithm for Maximum Matchings in Bipartite Graphs. *SIAM Journal on Computing* **2:** 225-231
- Jeong H, Mason SP, Barabashi AL, Oltvai ZN (2001) Lethality and contrality in protein networks. *Nature* **411**: 41-42.
- Kotlyar M, Fortney K, Jurisica I (2012) Network-based characterization of drug-regulated genes, drug targets, and toxicity. *Methods (San Diego, Calif)* **57:** 499-507
- Liu Y-Y, Slotine J-J, Barabási A-L (2011) Controllability of complex networks. *Nature* **473:** 167-173
- Hase T, Tanaka H, Suzuki Y, Nakagawa S, Kitano H (2009) Structure of protein interaction network and their implications on drug design. *PLoS Computational Biology* **5**: e1000550.
